# The Level of Community Integration in Adults with Psychiatric Disorders

**DOI:** 10.1192/j.eurpsy.2025.481

**Published:** 2025-08-26

**Authors:** E. D. Cindik-Herbrueggen

**Affiliations:** Psychiatry, Neuro-Psychiatric Center, München, Germany

## Abstract

**Introduction:**

In the post-migration period, individuals from diverse religious, linguistic, cultural, and traditional backgrounds strive to coexist in a shared social space. However, numerous adaptation challenges arise during this process. Additionally, individual personality traits significantly influence experiences during this time. For those suffering from psychiatric disorders, the integration process becomes even more complex, as these conditions can diminish an individual’s ability to cope with stress, making societal integration more difficult. This study aims to examine the level of integration among individuals with psychiatric disorders who have faced challenges after migration, with the goal of contributing to initiatives that facilitate their adaptation process.

**Objectives:**

This study aims to assess the integration levels of individuals with psychiatric disorders into society. In addition to examining psychiatric conditions, sociodemographic data will also be collected from participants to explore how additional factors influence the integration process. By doing so, the study seeks to provide insights that can inform efforts to facilitate the integration of these individuals into society.

**Methods:**

Data were collected from 91 participants, primarily first- and second-generation Turkish immigrants with at least one psychiatric disorder, at the Neuro Psychiatric Center Riem (NPZR) clinic. The study employed the “Community Integration Scale for Adults with Psychiatric Disorders” along with a sociodemographic questionnaire, with informed consent obtained from all participants. SPSS analysis software was utilized to examine the relationship between integration levels and other relevant variables.

**Results:**

Statistical analyses indicate that variables such as age, education level, smoking, and drug use significantly affect the total score.

**Conclusions:**

These findings reveal that individuals’ demographic and behavioral characteristics play a significant role in overall performance and achievement measures.

**Disclosure of Interest:**

None Declared

